# PFAS-Free Energy Storage: Investigating Alternatives for Lithium-Ion Batteries

**DOI:** 10.1021/acs.est.4c06083

**Published:** 2024-12-04

**Authors:** Eleni K. Savvidou, Amanda Rensmo, Jonathan P. Benskin, Steffen Schellenberger, Xianfeng Hu, Marcel Weil, Ian T. Cousins

**Affiliations:** † Stockholm University, Department of Environmental Science, SE-106 91 Stockholm, Sweden; ‡ RISE Research Institutes of Sweden, Environment and Sustainable Chemistry Unit, SE-114 28 Stockholm, Sweden; § SWERIM AB, Aronstorpsvägen 1, SE-974 37 Luleå, Sweden; ∥ Helmholtz Institute Ulm for Electrochemical Energy Storage (HIU), 89081 Ulm, Germany; ⊥ Institute for Technology Assessment and Systems Analysis (ITAS), Karlsruhe Institute of Technology, 76021 Karlsruhe, Germany

**Keywords:** fluoropolymers, PVDF, renewable energy, green energy transition, cathode, binder, electrolyte salt, electrolyte additives

## Abstract

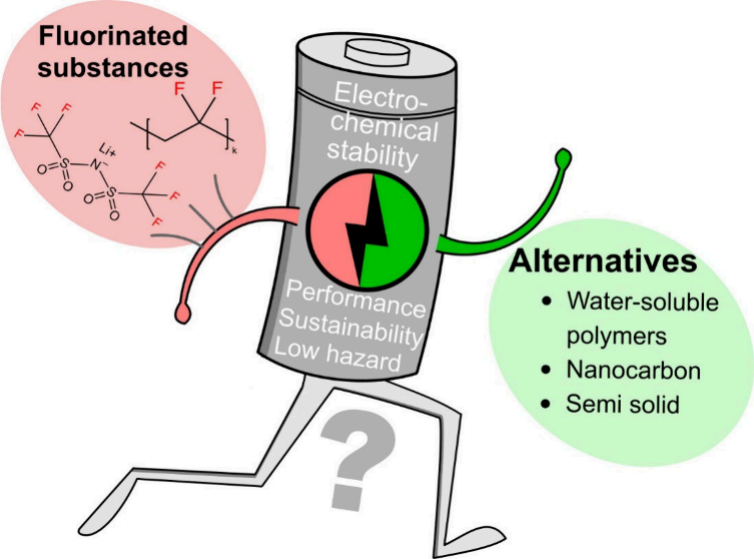

The class-wide restriction proposal on perfluoroalkyl and polyfluoroalkyl substances (PFAS) in the European Union is expected to affect a wide range of commercial sectors, including the lithium-ion battery (LIB) industry, where both polymeric and low molecular weight PFAS are used. The PFAS restriction dossiers currently state that there is weak evidence for viable alternatives to the use of PFAS in LIBs. In this Perspective, we summarize both the peer-reviewed literature and expert opinions from academia and industry to verify the legitimacy of the claims surrounding the lack of alternatives. Our assessment is limited to the electrodes and electrolyte, which account for the most critical uses of PFAS in LIB cells. Companies that already offer or are developing PFAS-free electrode and electrolyte materials were identified. There are also indications that PFAS-free electrolytes are in development by at least one other company, but there is no information regarding the alternative chemistries being proposed. Our review suggests that it is technically feasible to make PFAS-free batteries for battery applications, but PFAS-free solutions are not currently well-established on the market. Successful substitution of PFAS will require an appropriate balance among battery performance, the environmental effects associated with hazardous materials and chemicals, and economic considerations.

## Abstract 2

Contrary to the battery industry’s claims, there are potential alternatives to the use of PFAS in lithium-ion batteries.

## Introduction

Efforts to substitute certain uses of per- and polyfluoroalkyl substances (PFAS) with PFAS-free alternatives are being opposed by both the fluorochemical industry and the manufacturers of renewable energy technologies.^1−5^ The arguments used by these industries are as follows: 1) that PFAS are essential for a green energy future, with no viable alternatives in sight; 2) that the European Union’s (EU’s) PFAS restriction proposal^6^ will inhibit innovation and economic growth; and 3) the implicit assumption that chemical regulation is less critical than climate mitigation. This Perspective examines these arguments and counterarguments for the continued use of PFAS in lithium-ion batteries (LIBs) and potential future battery technologies. Modern society increasingly relies on LIBs for energy storage in, for example, electronics (laptops, cell phones, tablets), toys, power tools, and electric vehicles, besides stationary applications. Given the increasing production volumes of LIBs,^7^ the demand for certain PFAS used in their manufacturing is also expected to rise. PFAS are recognized for their persistence and widespread occurrence in the environment,^8^ with some linked to concerning health effects.^9^

In our recent review paper on PFAS in LIBs,^10^ we noted that the EU’s PFAS restriction proposal^6^ includes a claim that PFAS-free alternatives for use in LIBs are currently unavailable. RECHARGE, Europe’s industry association for advanced rechargeable and lithium batteries, recently reviewed and explained (in an online document^2^) the types of PFAS used in LIBs. RECHARGE also considered the availability of non-PFAS alternatives for multiple identified uses of PFAS in LIBs. They concluded that there are presently no viable alternatives available for the use of PFAS in LIB electrodes and electrolytes. The German Electro and Digital Industry Association ZVEI has made similar claims.^11^ In light of the ongoing consultation on the PFAS restriction proposal, we decided that it was necessary to carefully and independently evaluate these claims.

For this Perspective, a search of the available scientific literature was conducted, including peer-reviewed journal articles, monographs, industry reports, product descriptions, and patents. In addition, we contacted some innovative electrode and electrolyte manufacturers and downstream users and received additional input from technical experts from both industry and academia. We acknowledge that PFAS (largely fluoropolymers) are used as separator coatings, gaskets/seals, pipes, valves and sealings,^1^ and considering alternatives to all of these uses of PFAS requires a more thorough investigation and have been excluded from the study for now.

## Findings from Our Review and Consultations with Industry and Academia

### Alternatives for the Electrodes

**Figure 1:**
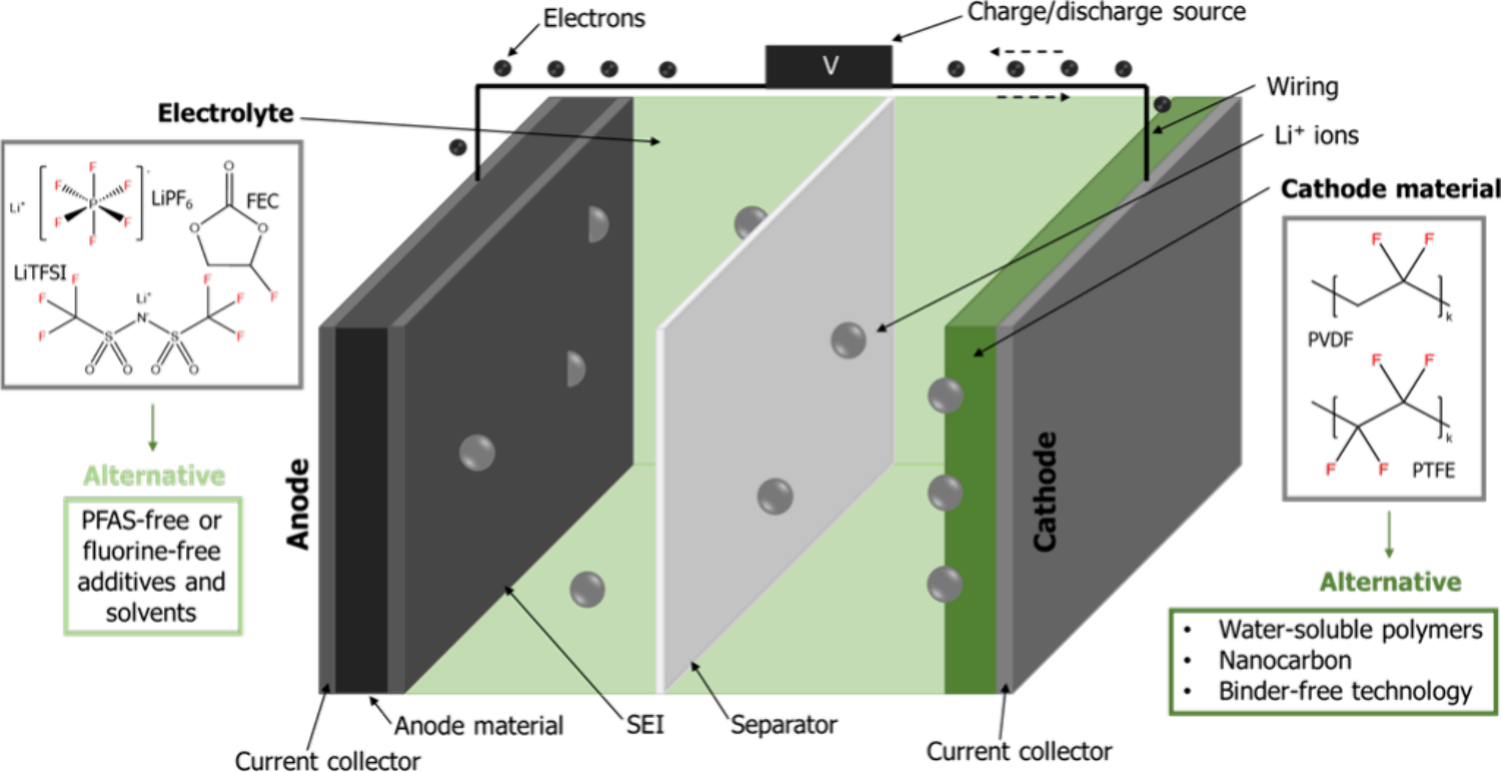
Schematic structure of a lithium-ion battery cell highlighting the components where PFAS (and other fluorinated substances) are used to the largest extent (with given example structures) and alternatives are needed. Adapted from ref (10) under the terms of a Creative Commons Attribution-NonCommercial 3.0 License. Copyright 2023 The Authors.

In order to bind the active material (electrochemically active components, e.g., lithium metal oxide) and make it adhere to the current collector, a polymeric binder is used in the electrode (Figure 1). The polymeric binder is essential for battery efficiency as it provides the electrodes with the necessary structure and robustness for effective electron movement and ion transition during the process of charging and discharging.^12^ In modern battery designs, the negative electrode (anode) made of graphite or silicone commonly uses nonfluorinated binders, e.g. carboxylmethyl cellulose (CMC), styrene–butadiene rubber (SBR), poly(acrylic acid) (PAA) and alginate.^13,14^ For the positive electrode (the cathode) polyvinylidene fluoride (PVDF) and variations such as polyvinylidene fluoride cohexafluoroethylene (PVDF-HFP), are commonly used due to the chemical inertness and high thermal stability derived from the carbon–fluorine bond as well as strong adhesive properties.^12^ In some cases blends of PVDF-copolymers and fluorinated ionic liquids are used because these ionic liquids provide useful antistatic properties.^15^ The manufacturing process of the cathode with PVDF as binder involves the use of *N*-methyl-2-pyrrolidone (NMP) which is a teratogenic solvent. According to RECHARGE, the only viable alternative involves using dry electrode processing, which eliminates the use of the toxic NMP, but requires application of polytetrafluoroethylene (PTFE) instead of PVDF. While PTFE is favorable for reducing occupational exposure to NMP, its lifecycle includes similar environmental impacts to PVDF.^16^ Modern fluoropolymer products are typically inert and contain few impurities, so in the use phase they are usually nonproblematic.^17^ However, considering the lifecycle of fluoropolymers the manufacturing and waste handling (especially using heat treatment processes with insufficiently high temperatures) can account for the emission of low molecular weight hazardous PFAS to the environment.^16,18−20^ Despite efforts to improve fluoropolymer manufacturing and waste handling in recent years,^21^ many problems remain. There are, for example, both regulated and nonregulated releases of multiple PFAS during fluoropolymer manufacturing,^18^ which can affect areas surrounding the plant.^22^ Moreover, there is growing evidence of PFAS emissions during waste handling,^16,23^ including during the recycling of LIBs.^10^

In the scientific literature, there are multiple reports of development of alternative materials to PVDF, which were initially motivated by replacing or abstaining from the use of NMP. Bresser et al.^24^ reviewed a wide range of alternative binders for sustainable electrochemical energy storage. They point out that PVDF is expensive and not environmentally friendly. Bresser et al.^24^ instead recommend using other materials such as water-processable PFAS-free polymers, which they claim could also reduce costs by a factor of 2–3 for the polymer and by a factor of about 100 for the processing solvent (NMP vs water). Innovation is, for example, ongoing to make an aqueous environment-friendly gelatin binder for LiFePO_4_ cathodes.^25^ Moreover, Rynne et al.^26^ showed that the commercially available elastomer Lotader 5500 (polyethylene-*co*-ethyl acrylate-*co*-maleic anhydride thermoplastic elastomer) could provide similar performance to PVDF in LIB composite electrodes with LiFePO_4_ and Li_4_Ti_5_O_12_ cathodes.^26^ However, this technology is applicable for coin cell batteries and still needs to be tested for industrially produced cylindrical or pouch cells as well as other cathode chemistries such as lithium nickel manganese cobalt oxides (NMC) which are more commonly used. Nguyen and Kuss^27^ reviewed the potential of a wide variety of conducting polymers as binders in LIBs, some (but not all) of which are PFAS-free alternatives to PVDF.^27^ Another study by Dobryden et al.^28^ examined biobased (natural organic polymer) binders which can be comprised of cellulose, lignin, alginate, gums, starch, and other biobased materials, and reviewed the current progress for these type of binders. They found that the raw materials have a rather low long-term performance which can be improved by surface modifications, but this approach still requires optimization to be commercially viable.^28^ In addition to organic (polymeric) binders, the application of ionically conducting inorganic binders was also explored recently by Trivedi et al..^29^ In that work, 12 binders based on sodium or lithium phosphates and silicates were examined for several different cathode materials, and shown to excel in performance compared to commonly used PVDF. These alternatives also demonstrated an improvement in battery manufacturing and recycling yields.^29^ The possibility for implementation of these novel technologies can vary depending on the cathode chemistry, and challenges for upscaling could arise for higher mass-loadings or different cell designs, among other aspects.

Although many of the breakthroughs in fundamental science surrounding LIB technologies stem from academia, the academic literature regarding future alternatives can be considered somewhat idealistic and does not by default provide a full indication of what is commercially viable and/or available on the market.^30^ This mismatch can be because 1) innovation occurs at companies as well as academia and can be trade secrets and 2) academic discoveries may work at the lab-scale but have not been scaled up and tested whether they are suitable for mass production. This also highlights the need for stronger cooperation between academic and industry research. Furthermore, economic applicability is an important deciding factor for industry, as facilities are designed for established manufacturing processes and changes would potentially result in high costs. We therefore sought to identify companies that already provide PFAS-free binder solutions, which are presented in the following paragraphs.

The company Leclanché has been using aqueous binders without organic solvents in its production process already for around 13 years.^31^ Their technology is used in LIBs for stationary energy storage (commercial and industrial) and e-mobility (heavier vehicles such as trains and buses). At the beginning of 2023, the company began the production of graphite anode and NiMnCoAl cathode cells with reduced cobalt content (as little as ∼5%) and a high nickel content of ∼90% using a water-based binder.^32^ Further, they have validated and produced PFAS-free electrodes, and with minor process adaptions, can now manufacture PFAS-free cells using the standard electrode stacking process.^33^ The company GRST has commercialized PFAS-free battery cells since 2022, with its proprietary water-based technology.^34^ Finally, the Centre for Solar Energy and Hydrogen Research Baden-Württemberg (ZSW) demonstrated pilot-scale production of water-based electrodes and cells that are free from NMP and fluorinated binders thus environmentally friendly in this aspect, using an alternative process that is cost-effective and suitable for mass production of cathodes.^35,36^

Despite their potential, water-soluble polymeric binder alternatives come with specific challenges, depending on the specific battery chemistry. As exemplified by the Leclanché alternative above, the market is shifting toward cathodes with a higher nickel content, driven by their overall superior performance.^37^ It is expected that chemistries of NMC 811 (LiNi_0.8_Mn_0.1_Co_0.1_O_2_, Ni-rich lithium nickel manganese cobalt oxide) or other chemistries with high Ni-content together with LFP (LiFePO_4_, lithium iron phosphate) will be the most prevalent lithium-ion batteries.^38^ However, a cathode chemistry with higher nickel content is more susceptible to processes involving water, which can lead to an increase of the pH of the slurry (suspension of active material). This can cause corrosion of the aluminum current collector, reducing the longevity and performance of the cell.^36^

Another potentially viable PFAS-free binder alternative for the cathode is a carbon-based material developed by Nanoramic Laboratories. This material, marketed as “Neocarbonix at the Core”, is a three-dimensional nanocarbon binding structure.^39^ This technology does not require NMP for solvent-processing or PFAS in binders and works as a drop-in replacement, allowing existing manufacturing processes and equipment to remain unchanged. Furthermore, it exhibits compatibility with a wide range of battery technologies.^40^ However, it is important to note that this technology is currently in its start-up stage.

Lastly, the companies 24M and FREYR produce binder-free electrodes, using a technology called “SemiSolid”, which is a mix of electrolyte and the active material that involves a simple and economical manufacturing process.^41,42^ Currently, we have not gathered more information about their processes and materials.

Our findings show that in the case of cathodes, some emerging technologies have been turned into commercially viable products or are close to this stage. This is in contrast to RECHARGE’s claim that the dry process using PTFE without NMP is the only viable alternative to PVDF in the cathode. A question that remains is if these technologies are suitable for large-scale production with competitive life cycle performance and thus can withstand growing demand in the near future in very different application fields with different technological performance requirements. Additionally, the environmental impacts of the alternative materials in their full lifecycle need to be assessed further to avoid “problem shifting”, e.g., regrettable chemical substitutions, less safety or shorter lifetime, higher climate impacts and low circularity.^43^

### Alternatives for the Electrolytes

The electrolyte is the medium in which the Li^+^ ions are transferred between the electrodes when charging/discharging the battery and is usually composed of a salt and a (organic) solvent. It is essential that the electrolyte is thermally and chemically stable, exhibits high Li^+^ ion conductivity and electronic insulation.^12^ Specific properties that the electrolyte salt (in this case a lithium salt) must demonstrate are low molecular weight and nontoxicity, electrochemical stability and the ability to form an effective interphase between the electrolyte and electrode.^12^ For the design of electrolytes, fluorination of the key components can help achieve optimal performance requirements for LIBs.^44−46^ The common electrolyte salt is lithium hexafluorophosphate (LiPF_6_, CAS 21324–40–3, not a PFAS) dissolved in a mixture of carbonates (Figure 1).^12^ LiPF_6_ offers several key benefits, including high ionic conductivity and oxidation stability, along with the ability to passivate the electrode (passivating means creating a protective layer, the solid electrolyte interphase (SEI) layer, on the surface of electrode to prevent it from reacting with other components in the battery throughout many cycles). However, LiPF_6_ hydrolyzes in the presence of moisture with subsequent formation of hydrofluoric acid (HF) which can lead to corrosion of the cell with implications for performance, longevity and safety of LIBs.^12^

Addition of PFAS salts such as lithium bis(trifluoromethanesulfonyl)imide (LiTFSI, CAS 90076–65–6, classified as a PFAS) can replace a small percentage of the LiPF_6_ in some electrolyte formulations. Typically, the quantity of these additives is ≤10% of the total weight or volume.^44^ In some specific cases, LiTFSI can be used as the major component of the electrolyte.^47^ The impetus for using such PFAS (and the so-called fluorinated ionic liquids) in the electrolyte is to improve overall cycling performance, but they are also more thermally stable, nonflammable, and less prone to HF formation, which provides a higher performing, longer-lasting and safer battery application overall.^12,44,48,49^

Based on input from consultation with experts, it is less common for PFAS salts like LiTFSI to serve as the primary electrolyte salt due to their potential to cause corrosion of the aluminum current collector^50^ and their higher associated costs. It is important to note that electrolyte formulations are often trade secrets and that it is difficult to estimate the exact amounts of certain substances used. In a recent study by Guelfo et al.^51^ commercially available LIBs from different brands were analyzed for bis-perfluoroalkyl sulfonimides (bis-FASIs), including LiTFSI (bis-FMeSI), lithium bis(pentafluoroethanesulfonyl)imide (LiBETI; bis-FEtSI; CAS 132843–44–8) and lithium bis(nonafluorobutanesulfonyl)imide (LiNFSI; bis-FBSI; CAS 119229–99–1). The total mass in the batteries for LiTFSI ranged from 7.2 ng to 35.6 mg. Further, as mentioned above, the study noted the potential blending of the TFSI anion paired with a different cation in the PVDF binder due to its antistatic properties.^51,52^ Emissions of these PFAS electrolyte salts and additives should be better controlled as they are widely detectable in the environment,^51,53,54^ along with the inorganic salts PF_6_^–^ and BF_4_^–^.^55^

Solvents in the electrolyte may also be fluorinated to render them nonflammable, prevent electrochemical oxidation and promote the formation of a more stable and lithium fluoride (LiF)-saturated SEI.^12^ Given that the formed SEI can be unstable, reducing the capacity of the cell,^56^ partially fluorinating the solvents can help enhance their compatibility with the lithium salt, and can aid in protecting the electrodes.^12^ For instance, silicone-containing anodes have higher energy densities, but are more sensitive regarding the charge–discharge-cycle, as the material breaks after a short time due to volume expansion and detaches from the current collector.^56^ The addition of a fluorinated solvent such as fluoroethylene carbonate (FEC) can help prevent this by decomposing and passivating the electrode surface as LiF is formed.^57^

Research is underway to develop fluorine-free electrolytes for LIBs. Despite the prevalence of fluorine-based options, there are several fluorine-free anions available, such as perchlorate (ClO_4_^–^), bis(oxalato)borate (BOB), tris(oxalate)phosphate, tetracyanoborate, and dicyanotriazolate.^58^ Among these alternatives, the primary drawback is their limited ability to passivate the aluminum current collector when compared to LiPF_6_. However, BOB has demonstrated the most promising outcomes.^58^ In a study by Hernández et al.^59^ where a fluorine-free electrolyte based on LiBOB and vinylene carbonate was shown to provide higher discharge capacity and a longer cycle life than a cell using a highly fluorinated electrolyte for an NMC111 (LiNi_0.33_Mn_0.33_Co_0.33_O_2_) cathode with silicon-graphite composite anodes. For instance, He et al.^60^ report an aqueous electrolyte system using a lithium salt/polymer complex for LiTi_2_(PO_4_)_3_/LiMn_2_O_4_ and TiO_2_/LiMn_2_O_4_ lithium-ion cell with promising results achieving energy densities up to 124 Wh/kg. It expands the possibilities of introducing nontoxic, high-conductivity, and dimensionally stable aqueous electrolytes, which enables the development of environmentally friendly, nickel-, cobalt-, and fluorine-free LIBs.^60^ Khan et al.^61^ explored fluorine-free electrolytes derived from biomass such as lithium furan-2-carboxylate dissolved in tetra(*n*-butyl)phosphonium furan-2-carboxylate. Examination of the physicochemical properties showed high thermal stability, acceptable ionic conductivities, and wide electrochemical stability.^61^

The electrolyte company E-Lyte has announced a collaboration with Nanoramic, which entails the development of a customized PFAS-free electrolyte for their alternative cathode technology.^62^ Currently no information is available regarding the alternative chemistries they intend to use, and it is likely that this will remain a trade secret, even after development. Electrolyte formulations are often customized and developed with a high degree of confidentiality by the electrolyte supplier depending on the needs of the battery manufacturer, who may be unaware of the precise composition. Therefore, the electrolyte suppliers could seemingly have the flexibility to adapt to developments of PFAS-free or even fluorine-free alternative electrolytes, depending on the favored alternative cathode chemistry. This could be the case if providing PFAS-free electrolytes results in a competitive advantage, as battery manufacturers would begin to request these formulations. But it seems that LiPF_6_ will still remain in the picture as the leading main salt due to its performance abilities. A study by Nam et al.^63^ developed a battery system using nonfluorinated alternatives such as an aromatic polyamid (APA) binder and lithium perchlorate (LC) electrolyte, which deliver comparable performance to traditional fluorinated components. This study shows a promising direction for developing fluorine-free battery systems.

In conclusion, most LIB systems can function with LiPF_6_ and a nonfluorinated solvent, but it is uncertain if this alone provides a sufficient level of performance and safety for all LIB applications. We have not been able to fully evaluate all the trade-offs between PFAS-containing and PFAS-free options in electrolytes, and this is an important activity for future research. A summary for the above-mentioned alternative binders and electrolytes are summarized in Table 1.

**Table 1:** Summary of PFAS Used in the Cathode and Electrolyte and Their Potential Alternatives

battery part	fluorinated substance	function	possible alternative	availability of alternative
Cathode	PVDF	Binder	• Water-soluble polymers	Commercially available
			• 3D nanocarbon binding structure (Neocarbonix at the Core)	
	PTFE	Binder	• Binder-free electrode (SemiSolid)	
Electrolyte	Salt additives and electrolyte components, e.g. LiTFSI	Commonly additives, less often as main electrolyte components	Nonfluorinated alternatives	Depending on overall cell chemistry electrolyte can be customized accordingly
	Solvents, e.g. FEC	Additive	Nonfluorinated alternatives	Depending on overall cell chemistry electrolyte can be customized accordingly

### Solid-State Batteries

Solid-state batteries, which use different kinds of solid-phase electrolytes, are widely considered to be the next generation of batteries close to market implementation.^64,65^ However, it is expected that a number of technical challenges must be overcome before solid-state batteries can be commercialized.^66^ RECHARGE claim that uses of PFAS, including PVDF and polytetrafluoroethylene (PTFE), will be even more important in solid-state batteries.^1^ As outlined in the review of Ahniyaz et al.,^66^ solid-phase electrolytes can be solid polymeric electrolytes, solid inorganic electrolytes, or intermediates between the two. There are also intermediates between solid and liquid electrolytes, which are the liquid-like gel polymer electrolytes. Among the multitude of material options reviewed in Ahniyaz et al.,^66^ both PFAS-free and PFAS-containing materials are under consideration for use in gel polymer and solid-phase electrolytes. Among the gel polymer electrolytes, PVDF-based materials are among the most widely used, but PFAS-free gel polymer electrolytes are also being developed.^66^ The majority of solid-phase polymer electrolytes in development are based on (PFAS-free) poly(ethylene oxide) (PEO), but fluoropolymer-based solid-phase electrolytes are also under consideration.^66^ For example, a PVDF-based solid-phase electrolyte was proposed by Zhang at al.,^67^ and a PVDF-HFP-based solid-phase electrolyte was proposed by Du et al.^68^ With regards to materials used in the electrodes of solid-state batteries, conducting polymer-based binders were suggested to provide excellent performance and nonfluorinated options are available (see review of Nguyen and Kuss).^27^

We conclude that the future is uncertain regarding the likelihood of innovating toward high-performing PFAS-free solid-state batteries. It is also clear that state-of-the-art LIBs will dominate the battery market for the foreseeable future.^38^

### Alternative Battery Chemistries

At present, many different Li alternatives are under investigation, including those based on K, Ca, Al, Zn, Mg, or Na. Among these alternatives, sodium-ion battery (SIB) systems are the most promising group and are already undergoing field tests for large-scale application for electric vehicles.^69^ For the cathode, there are three main types of materials suitable for commercializing SIBs, comprising layered transition metal oxides (LTMO), polyanionic materials and Prussian blue analogs.^70,71^ The current state of research regarding these technologies implies the use of fluorochemicals in electrodes and electrolytes, similar to LIBs. Zheng et al.^72^ describe the broad similarities between LIBs and SIBs in terms of fluorinated substances added to electrolytes. Among others, the authors mention the use of TFSI-salts (PFAS) as electrolyte additives in SIBs, which give similar effects of thermal stability, higher ionic conductivity, and solubility. Similarly, FEC (not a PFAS) is recommended as an additive for the electrolyte also for SIBs in small amounts of up to 5 wt % for, among other benefits, its gas evolution suppression and ability to create stable SEI layer.^72,73^ Furthermore, Hernandez et al.^58^ discuss the increased ability to passivate the anode by adding fluorinated species to either LIBs or SIBs.

The company Altris has developed a SIB that uses Prussian white (Na_2_Fe[Fe(CN)_6_]·zH_2_O) for the cathode and sodium bis(oxalate)borate (NaBOB) as an electrolyte salt, achieving an energy density that can compete with common lithium-ion chemistry.^74^ Prussian white offers cost-effectiveness, sustainability, and good electrochemical performance.^75^ Meanwhile, NaBOB demonstrates durability over extended cycles and exhibits a high decomposition temperature.^76^

The similarity between the two battery chemistries and the proven benefits from fluorinated electrolyte chemistries in LIBs point to the possible use of PFAS also within the emerging alternative technology of SIBs. Mapping of PFAS within SIBs should be performed, as was previously done for LIBs,^10^ for a full understanding of the potential risks associated with these alternative battery chemistries.

### Implications for the Recycling of Batteries

The EU Batteries Regulation states that its main goal is to ensure increased sustainability, circularity and safer batteries on the European market.^77^ Furthermore, it mandates that a recycling efficiency of 65% by average weight of lithium-based batteries must be achieved by 2025, and 70% by 2030.^77^ The recovery rates for lithium are expected to be 50% by 2027 and 80% by 2031.^78^ It is therefore crucial to minimize the use of toxic substances within LIB materials in order to improve recyclability and reduce the number of process steps.

The occurrence of fluorine as well as PFAS in LIBs pose numerous challenges during the recycling process.^79^ In addition to requiring more steps for removal (and by extension, additional cost and lower yields), the generation of corrosive HF can damage equipment^80^ and represents a significant occupational health risk. Moreover, inorganic and organic fluorinated byproducts formed during recycling (e.g., PFAS)^10^ may be released in recycling waste streams, and are problematic for the environment.^80,81^ Our view, based on research on incineration of PFAS,^82^ is that although pyrometallurgical recycling is energy-intensive, these processes (utilizing temperatures of up to 1600 °C) will be sufficient to mineralize PFAS (e.g., PVDF or PTFE used in electrode binders but also LiTFSI).^83^ During the recycling process, various fluorinated compounds, both inorganic (e.g., lithium fluoride, silicon tetrafluoride, phosphorus pentafluoride) and organic fluorinated species (e.g., perfluoroalkyl chains and fluorinated aromatic compounds), could be generated.^10^ These high temperatures facilitate the breakdown of fluorinated compounds into their mineral forms (i.e., carbon dioxide and fluoride), significantly reducing the likelihood of harmful PFAS emissions. On the contrary, the now-preferred hydrometallurgy process (without pyrometallurgical preprocessing) that operates at lower temperatures, involves a mechanical preprocessing step, and generates higher yields of the metals could inadvertently lead to increased PFAS emissions. When it comes to the recycling goals as stated in the EU Batteries Regulation, they might be only achievable using hydrometallurgy. Alternatively, closed loop recycling, which involves direct regeneration of the cathode materials,^84^ shows promise but is challenged by the necessary binder removal a challenge.^85^ This technology remains the subject of ongoing research. In any case, the recycling processes need optimization in order to close the gap between generating sufficient yields of the metals and reducing or avoiding potential PFAS emissions.

In conclusion, it would be beneficial to reduce the fluorinated substance content in batteries, in order to gain more purified recycled materials for battery manufacturing but also to keep the environmental impact low from these processes as well as to maintain a safe work environment. With this in mind, it is also pertinent to note that the alternative cathode technologies described above are more suitable for recycling compared to PFAS-containing materials in the electrode and electrolyte. Still, comprehensive technological and environmental lifecycle assessments might be needed for a quantitative comparison considering the full life cycle of a battery.

## Finding the Balance between Performance and Sustainability in the Transition to PFAS-Free Battery Technologies

Given the regulatory pressure on the entire class of PFAS, the battery industry will need to find a viable commercial alternative for PVDF in the cathode. Even though there are potentially suitable alternatives on the market in limited applications, adoption of these alternatives more widely may or may not reduce the performance of LIBs in certain applications. LiPF_6_ will probably continue to account for the majority of the electrolytes in LIBs in the near future. Although the addition of PFAS salts is not essential to the functioning of batteries, their presence may contribute to increased performance and safety. The requirements for battery materials are high as they need to be electrochemically stable and cope with high-voltage cycling. We also recognize that LIB performance requirements vary widely depending on their applications. However, there is the potential to have PFAS-free batteries in the future (with an estimated transition time of 7–10 years), according to the experts we consulted. Lastly, we acknowledge that the costs of the transition remain unclear and are something that requires further investigation.

In deciding on the favored technology options for the development of batteries, a balance needs to struck between the performance of future batteries and the sustainability of materials used (e.g., considerations of the lifecycle impact of materials and chemical components). It is not a trivial problem to balance these two issues because it requires detailed knowledge from multiple experts of different fields. We also realize the importance of batteries in the so-called “Green Energy Transition” and understand that innovation in this area should not be impeded. However, we should be cautious of giving actors in the renewable energy sector too much freedom in order to avoid the risk of “problem shifting”, i.e., replacing one environmental problem (e.g., climate change) with another (irreversible chemical pollution). Chemical pollution is considered by many to be a lesser environmental problem compared with climate change. This may well be true, but compared to climate change, the sources, impacts, and solutions of chemical pollution are less studied and understood. We should therefore not be complacent given the threats chemical pollution has to human health and biodiversity loss.

We find it surprising that companies providing green energy technologies are now arguing strongly and publicly to continue using hazardous chemicals such as PFAS in their products rather than focusing their efforts on possible alternatives. We would hope that green energy storage providers, which enjoy a green profile with many in the public, should also have pollution control as one of their core corporate principles. The PFAS restriction can be an opportunity for the European battery industry to become the frontrunner in revolutionizing energy storage systems toward true sustainability to benefit the environment as well as occupational safety, along with securing the energy and materials supply within Europe.

In addition, to ensure that sustainable materials and chemicals are used in the manufacture of batteries, it is also important to have functioning recycling processes. The service life of LIBs is in the range of 5–15 years depending on application, but it may take up to 20 years before end-of-life batteries are recycled. This means that even if PFAS-free batteries dominate the market in the future, there will be a need to recycle PFAS-containing batteries currently on the market, or entering the market, many years into the future. We call again for research to be conducted on the release of PFAS during battery recycling. In our view, the battery manufacturing industry, which is rapidly expanding, has the responsibility to fund and support such research. This should be accompanied by related national and international research calls to support the development of suitable innovative recycling processes on an industrial scale.

To provide a roadmap for effectively managing this issue going forward, we suggest the following three steps. Step 1 is the investigation of potential emissions from battery manufacturing and recycling. While some studies have detected PFAS used in LIBs in the environment, it remains unclear what emissions are directly linked to battery manufacturing and recycling. Step 2 focuses on identifying which specific PFAS are being emitted during manufacturing and recycling, the quantities of these PFAS being emitted, and assessing the associated risks. Finally, in Step 3, if unacceptable risks are identified, measures should be taken to adapt manufacturing and recycling processes to mitigate emissions, including exploring PFAS substitution as an “upstream” solution and treatment solutions as a “downstream” solution (i.e., capture and destruction technologies) for battery recycling processes and already contaminated sites.
